# Protein Splicing: How Inteins Escape from Precursor Proteins*

**DOI:** 10.1074/jbc.R113.540310

**Published:** 2014-04-02

**Authors:** Kenneth V. Mills, Margaret A. Johnson, Francine B. Perler

**Affiliations:** From the ‡ Department of Chemistry, College of the Holy Cross, Worcester, Massachusetts 01610,; the § Department of Chemistry, University of Alabama at Birmingham, Birmingham, Alabama 35294, and; ¶ New England Biolabs, Inc., Ipswich, Massachusetts 01938

**Keywords:** Enzyme Kinetics, Enzyme Mechanisms, Hedgehog, Post-translational Modification, Protein Motifs, Asparagine Cyclization, Bacterial Intein-like Domain, Intein, Protein Splicing, Thioester

## Abstract

Inteins are nature's escape artists; they facilitate their excision from flanking polypeptides (exteins) concomitant with extein ligation to produce a mature host protein. Splicing requires sequential nucleophilic displacement reactions catalyzed by strategies similar to proteases and asparagine lyases. Inteins require precise reaction coordination rather than rapid turnover or tight substrate binding because they are single turnover enzymes with covalently linked substrates. This has allowed inteins to explore alternative mechanisms with different steps or to use different methods for activation and coordination of the steps. Pressing issues include understanding the underlying details of catalysis and how the splicing steps are controlled.

## Introduction

**FIGURE 1.**
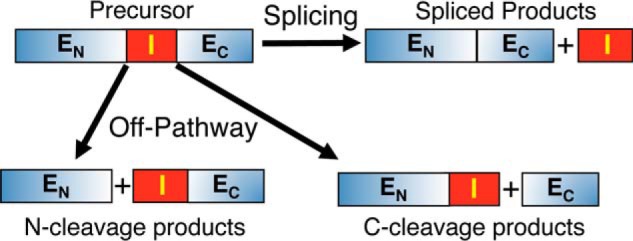
**Potential intein reactions.** Protein splicing results in ligation of the N-extein (*E_N_*) and C-extein (*E_C_*), as directed by the intein (*I*). When inteins are mutated or inserted in heterologous contexts, off-pathway reactions can occur resulting in N-terminal, C-terminal, or double cleavage products that are unable to splice. Off-pathway N-terminal cleavage can occur in both the linear and the branched (thio)ester intermediates. Off-pathway C-terminal cleavage occurs when cyclization of the intein C-terminal residue precedes branch intermediate formation.

The first intein sequence was published 25 years ago (1). In those early days of gene analysis, it was hard to decipher why the *Saccharomyces cerevisiae Sce VMA1* vacuolar ATPase gene was so large. Two years later two groups showed that a section of the *VMA1* gene was absent in the mature ATPase (2, 3). They challenged existing fundamental beliefs about gene expression by predicting that an internal section of this protein was removed by protein splicing instead of RNA splicing and that a single gene encoded two stable proteins: the host protein (extein) and the intervening protein (intein) (4). All attempts to demonstrate RNA splicing failed. *In vivo* time courses suggested protein splicing (5, 6), which was definitively established as a new method of gene expression when the elusive precursor protein was isolated by cloning a *Pyrococcus* species DNA polymerase intein between two unrelated proteins, resulting in temperature-dependent splicing (7). This first example of *in vitro* splicing revealed important mechanistic insights: splicing occurs when the intein and the first C-extein residue are embedded in a heterologous host protein, inefficient splicing can result in off-pathway single splice site cleavage (Fig. 1), splicing can be controlled, and splicing proceeds through a slowly migrating branched intermediate with two N termini.

**FIGURE 2.**
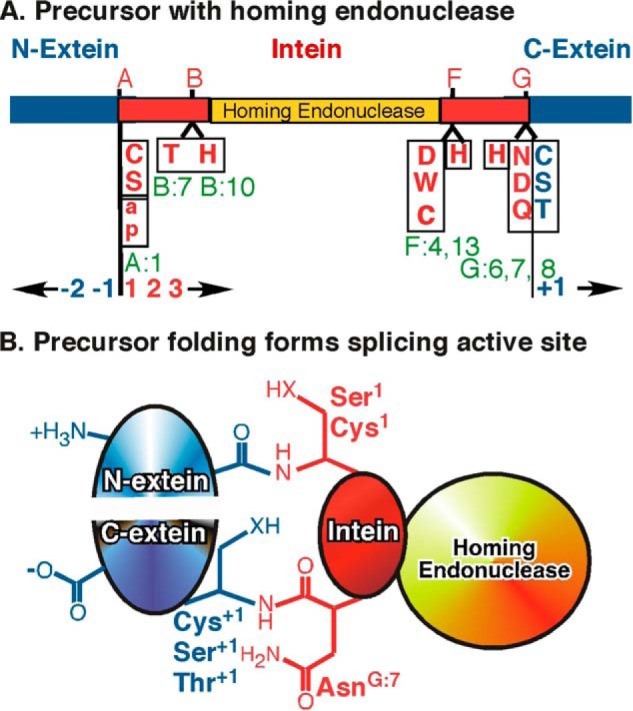
**Precursor domains and conserved motifs.** *A*, a precursor with an intein containing a homing endonuclease domain (*gold*) is depicted with intein splicing domain motifs (*red*) listed above and conserved residues that participate in catalysis listed below. Residues in intein motifs are numbered based on their position within each motif (*green*) as defined in InBase (20). Residues specific to class 2 or 3 inteins are in *lowercase*, and only a subset of residues found at A:1 is depicted. Motifs A, B, F, and G have also been called N1, N2, C2, and C1, respectively (17–20). Motifs C, D, E, and H are specific to certain homing endonucleases and are not shown. To simplify discussion of inteins in various precursors, residues in each part are numbered independently. Intein residues are numbered from the N to C terminus beginning with 1. Residues in the N- and C-exteins (*blue*) are numbered from the splice site outwards and include a *minus sign* for N-extein and a *plus sign* for C-extein residues. *B*, folding of the precursor forms the intein active site and initiates protein splicing. Homing endonuclease domains in larger inteins fold separately from the intein and extein domains. Association of extein fragments can influence precursor folding and active site architecture. *X* represents an oxygen or a sulfur atom.

We consider inteins to be single turnover enzymes because they use the same strategies as classical enzymes to perform catalysis (8). Splicing occurs in the absence of any known cofactor, chaperone, or energy source. All that is required is proper folding of the intein in the precursor to align nucleophilic residues and residues that assist catalysis (Fig. 2), leading some to call inteins nature's escape artists (9). Because inteins utilize groups of similar nucleophiles, subtle variations in the reactivity of these amino acids require different sets of assisting residues. As a result, some residues facilitating catalysis may still be unknown.

**FIGURE 3.**
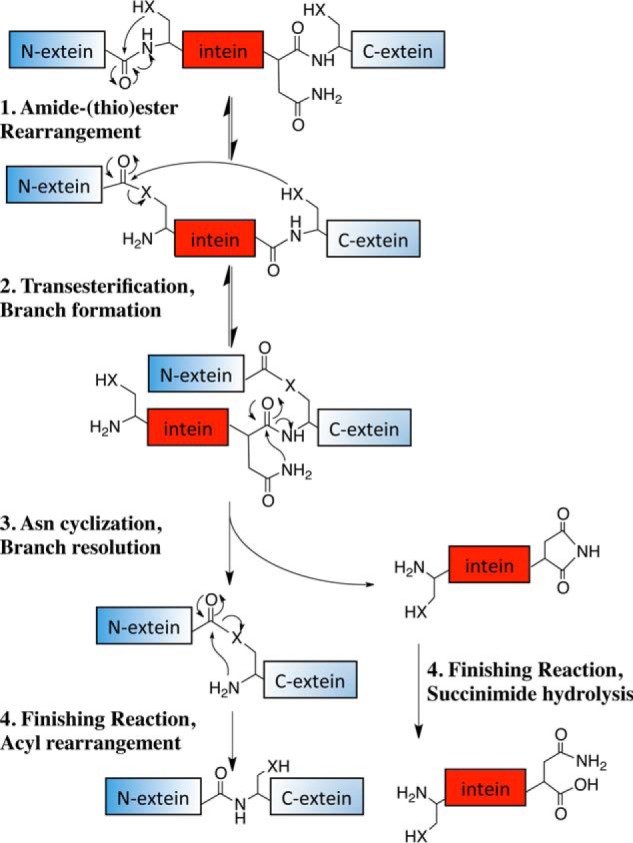
**The intein-mediated class 1 protein splicing mechanism.** Class 1 inteins with a C-terminal Asn and a Cys, Ser, or Thr at the first position in both the intein and the C-extein splice using the standard four-step protein splicing mechanism depicted in this figure. Inteins with C-terminal Glu, Gln, or Asp use this same mechanism except for Glu, Gln, or Asp cyclization in step 3, although other mechanisms are possible. Succinimide hydrolysis can also produce iso-Asn. *X* represents an oxygen or a sulfur atom. For clarity, tetrahedral intermediates and residues facilitating each step are omitted. Although the definition of an intein is the excised sequence (4), for brevity we will include the C-extein nucleophile when discussing mechanisms.

There are three classes of inteins based on sequence signatures and splicing mechanisms (10). The standard class 1 intein splicing mechanism (Fig. 3) consists of 1) an acyl rearrangement to convert the N-terminal splice site peptide bond from an amide to a (thio)ester, 2) a transesterification to form a branched intermediate, 3) Asn cyclization resolving the branched intermediate by cleaving the C-terminal splice site, and 4) a second acyl shift to form an amide bond between the ligated extein segments (5–7, 11–16). Off-pathway cleavage occurs when coordination of the steps is perturbed by mutation or by expression between foreign exteins (Fig. 1). This may result from an increase in the cleavage rate at that splice site, a decrease in the reaction rate of another step, or interference with a mechanism-linked conformational change required to promote a downstream step.

As the number of sequenced inteins increased, sequence alignments revealed four splicing motifs termed blocks A, B, F, and G (Fig. 2) (17–20). Although not conserved in their entirety, several positions in each motif contain highly conserved groups of similar amino acids. The nucleophiles for each step are: Cys^1^ or Ser^1^ in step 1; Cys^+1^, Ser^+1^, or Thr^+1^ in steps 2 and 4; and the intein C-terminal Asn^G:7^ in step 3 (see Fig. 2 for residue nomenclature). Known assisting residues include positions 7 and 10 in block B (Thr^B:7^ and His^B:10^), the intein penultimate His^G:6^, and the less conserved positions 4 and 13 in block F (Fig. 2). Position F:4 is most commonly Asp, followed by Cys and then Trp, and F:13 is most commonly His (10, 20).

Inteins come in many flavors. Most inteins are large chimeras containing both a splicing domain and the same type of endonucleases that mediate intron mobility (Fig. 2) (20–22). Other inteins are naturally occurring mini-inteins that are as small as 134 residues and lack an endonuclease domain (20). Studies of both native and engineered mini-inteins helped define the intein splicing domain (20, 23–27). Intein genes may also be split between motifs B and F; however, the expressed precursor protein fragments rapidly assemble to splice in *trans* by the same mechanisms used in *cis*-splicing inteins (28–32). Both naturally occurring and engineered split inteins have found great utility in biotechnology applications (33). Intein splicing domains may have been derived from ancient enzymes because they are small and are closely related by structure, conserved motifs, and enzymatic activities to Hedgehog autoprocessing domains, which activate essential signaling proteins for metazoan development (34, 35). Inteins are also related to bacterial intein-like (BIL)^2^ domains (36–39).

Establishing a universal mechanism for all inteins at the atomic level is unlikely because the methods used to promote each rearrangement and the roles played by the assisting residues vary. Instead, we will discuss both the canonical and the alternative protein splicing mechanisms, and general strategies for catalysis and coordination of the steps. Detailed mechanism reviews are available (8, 9, 40), and intein structure, molecular dynamics, evolution, and applications are covered in companion reviews in this series (21, 33, 41).

## The Class 1 Splicing Mechanism: Step 1

Conversion of amide bonds to enzyme-linked thioesters or esters, as in step 1 of protein splicing, is a common method of catalysis used by proteases and autoprocessing enzymes including glycosyltransferases and pyruvoyl enzymes (8, 9, 40). Although protein splicing employs a series of bond rearrangements rather than the bond cleavage facilitated by proteases, inteins use similar strategies to destabilize the peptide bond to favor (thio)ester formation, including catalytic bond strain, general acid/base catalysis, and an oxyanion hole to stabilize the tetrahedral intermediate.

Most residues involved in catalysis assemble near the center of the disk-shaped HINT (Hedgehog-intein) fold of the intein splicing domain (34). For example, residues near the N-terminal scissile bond include Thr^B:7^ and His^B:10^ in a type I β-turn and Asp^F:4^ in a β-strand. Experimental data show that Thr^B:7^, His^B:10^, and residue F:4 affect N-terminal splice site reactions, as do flanking extein residues (13, 14, 20, 42–51). His^B:10^ is the most conserved intein residue (20). Of the two inteins without His^B:10^, one is a degraded pseudogene (52), and the other (the *Thermococcus kodakaraensis* Tko CDC21-1 intein) uses Lys^58^ to activate the N-terminal splice site by possibly stabilizing the initial N–S acyl shift tetrahedral intermediate (53). Lys^58^ lies outside the conserved intein motifs (53) and is one residue beyond a newly identified position (22 residues past His^B:10^) that potentially activates the N-terminal nucleophile (40). N-extein residues were shown to influence the equilibrium position between amide and ester in the Sce VMA intein (44) and to affect N-terminal reactions by van der Waals contacts with *Pyrococcus horikoshii* Pho RadA intein residues (46). The *Nostoc punctiforme* Npu DnaE intein +2 C-extein residue also affects splicing, possibly by filling space at the active site to optimally align catalytic residues (48, 49).

Some inteins distort the N-terminal scissile bond generating catalytic strain to accelerate step 1. Adjacent extein residues and block B residues help form this strained local conformation, as evidenced by both structural and biochemical studies. A crystal structure of the Sce VMA intein displays bond angle distortions near the N-terminal splice junction (54). The *Mycobacterium xenopi* Mxe GyrA intein crystal structure has a *cis*-peptide bond linking the N-extein and intein, and NMR data suggest a lack of amide bond resonance that is resolved when His^B:10^ is mutated (42, 55). His^B:10^ is in hydrogen bond distance to the amide nitrogen of the N-terminal scissile bond in several inteins, suggesting that it plays a role in coordinating the scissile bond (54–57). A similar role was observed for Thr^B:7^ in a *Synechocystis* species Ssp DnaE intein structure (58).

A second strategy is to accelerate the rate at which the amide-ester equilibrium is reached by activating the N-terminal nucleophile via thiol deprotonation (experimentally detected as a lower p*K_a_*), stabilizing the tetrahedral intermediate, and/or influencing how the tetrahedral intermediate is resolved. Likely contributors again include Thr^B:7^, His^B:10^, and Asp^F:4^. A close look at the *Mycobacterium tuberculosis* Mtu RecA intein provides several lines of evidence to support these strategies. NMR and quantum mechanical/molecular mechanics studies suggest that His^B:10^ may deprotonate the thiol of the N-terminal Cys^1^ to drive formation of the tetrahedral intermediate and then donate the proton to the Cys^1^ α-amino group to resolve the tetrahedral intermediate as a thioester (59). These roles are supported by changes in the p*K_a_* of His^B:10^ from neutral to acidic during splicing (60). Similarly, a Mtu RecA intein structure shows Asp^F:4^ in position to hydrogen bond to the thiol of Cys^1^ to deprotonate this nucleophile (61). Moreover, the p*K_a_* of the Mtu RecA intein Asp^F:4^ is elevated and the p*K_a_* of Cys^1^ is lower than normal, but both p*K_a_* values return to normal when either residue is mutated (59). Taken together, these studies support a proposed proton transfer network in the Mtu RecA intein that assists deprotonation of the Cys^1^ nucleophile and the forward resolution of the tetrahedral intermediate (59–61). However, studies of the *Synechocystis* sp. PCC6803 Ssp DnaB intein with an unnatural N-terminal residue indicate that activation of this nucleophile is not essential for linear thioester formation (62), emphasizing the unique active sites and catalytic strategies utilized by individual inteins.

## The Class 1 Splicing Mechanism: Step 2

The second step has proven the most challenging to study as it is difficult to isolate branched intermediates. Mutations that should result in accumulation of branched intermediates often result in decay to N-terminal cleavage products, especially when a thioester linkage is present (13). Ester-linked branched intermediate formation is reversible, which can result in accumulation of precursor rather than intermediate (7). Several studies suggest that the intein promotes step 2 by controlling the protonation state of the +1 nucleophile. For example, the p*K_a_* of Cys^+1^ in the Mtu RecA intein is depressed to 5.8, increasing its nucleophilicity at physiological conditions (63). Furthermore, quantum mechanical simulations suggest that Cys^+1^ in the Mtu RecA intein may be deprotonated by Asp^F:4^ and that this deprotonation may be driven in part to stabilize the positive charge on the α-amino group of Cys^1^ in the linear thioester intermediate (64). Step 2 is strictly coupled to step 1 in class 1 inteins, although the exact mechanism has yet to be determined (13–15, 65). It is possible that linear thioester formation removes elements that are masking the reactive thiol of the +1 residue or induces a conformational change to align active site residues for transesterification (see below).

## The Class 1 Splicing Mechanism: Step 3

Evidence for the third step of splicing includes loss of C-terminal splice site cleavage after mutation of the intein C-terminal Asn^G:7^ and the detection of excised inteins with C-terminal succinimide residues (11–15). The intein must catalyze Asn cyclization, because in other systems it results in side-chain deamidation rather than peptide bond cleavage (66), and computational modeling suggests very high energy barriers in non-catalyzed models of cleavage by Asn cyclization (67).

Several strategies have been proposed for enzymatic activation of step 3 including three coupled modes of catalysis: 1) His^F:13^ increases the nucleophilicity of the C-terminal Asn^G:7^ by deprotonation, 2) the tetrahedral intermediate is stabilized by charged His^F:13^ and His^G:6^ residues, and 3) the electrophilicity of the backbone amide may be increased by His^G:6^ (55, 57, 67–69). Alternatively, given that C-terminal cleavage is favored at low pH (65, 70, 71), protonation of the backbone amide nitrogen of the scissile peptide bond may have precedence over deprotonation of the Asn side-chain amide (72). Separate studies suggest two other modes of catalysis: change in the local environment near the scissile bond that depends on branched ester formation (69) and destabilization of the scissile bond by a polarizable adjacent C-extein residue (73).

His^F:13^ and His^G:6^ are not required for Asn cyclization in all inteins (28, 56, 74, 75). Mutation of His^F:13^ in a class 2 intein had no effect (76), and ∼5% of functional inteins have an alternate G:6 residue (20, 28, 56, 74, 75). Splicing can be enhanced by “reverting” back to His^G:6^ in some inteins, whereas a His^G:6^ actually impairs splicing in other inteins (28, 74, 75, 77). These differences may reflect different positions along the evolutionary path to overcoming loss of His^G:6^.

Some inteins lacking Asn^G:7^ have similar residues (Asp and Gln) that can undergo cyclization to cleave the C-terminal splice site (20, 71, 78–80). For both the *Pyrococcus abyssi* and the *Methanoculleus marisnigri* Pol II inteins, splicing with a C-terminal Gln is slow, but is improved with substitution to Asn (71, 78, 79). On the other hand, the *Chilo* iridescent virus ribonucleotide reductase (CIV RNR) intein can splice with a native C-terminal Gln more efficiently than with Asn (80). As in the case of inteins lacking His^G:6^, it is likely that these variant inteins represent different stages in evolving optimal activity after an initial mutation removed a catalytically important residue. Retaining a slow or inefficient step 3 may not be detrimental when it does not lead to off-pathway N-terminal cleavage.

## The Class 1 Splicing Mechanism: Step 4

Step 4 consists of two finishing steps, neither of which is necessarily catalyzed by the intein. The intein C-terminal aminosuccinimide is slowly hydrolyzed to Asn or iso-Asn (11–13), and the (thio)ester linking the extein segments reverts to the amide. Experiments with model peptides demonstrate that the rate of conversion from a (thio)ester to an amide is faster than the overall rate of splicing (81). This final acyl shift is thermodynamically favorable and is not influenced by the presence of the intein (11, 69).

## Variant Splicing Mechanisms: Class 2 Inteins, Class 3 Inteins, and BILs

**FIGURE 4.**
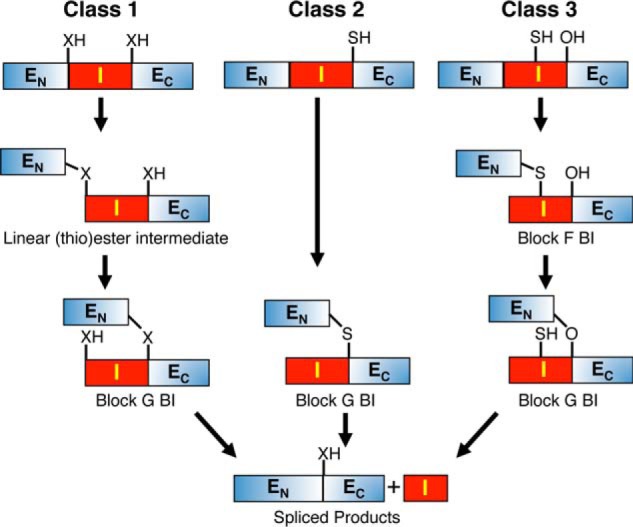
**Variations in splicing mechanisms.** Inteins missing the standard N-terminal nucleophile use various strategies to get to the same block G branched intermediate formed after step 2 in class 1 inteins. Class 2 inteins form the block G branched intermediate after direct attack on the amide bond at the N-terminal splice site by Cys^+1^. Class 3 inteins first form a block F branched intermediate with Cys^F:4^ as the branch point and then transfer the N-extein to the +1 residue to form the block G branched intermediate. Once the block G branched intermediate is formed, class 2 and class 3 inteins follow the same steps (3 and 4) to complete splicing as in class 1 inteins. Abbreviations used are: *E_N_*, N-extein; *E_C_*, C-extein; *I*, intein; *BI*, branched intermediate; *X*, an oxygen or a sulfur atom.

The robustness of intein-mediated protein splicing is illustrated by the array of acceptable modifications to the standard four-step mechanism. BILs lack the C-extein +1 nucleophile and are therefore unable to form the block G branched intermediate (36–38). Both class 2 and class 3 inteins can still splice, although they lack a Ser^1^ or Cys^1^ nucleophile and are thus unable to form the linear (thio)ester intermediate (Fig. 4).

To date, all class 2 inteins are orthologs of the *Methanococcus jannaschii Mja KlbA* intein (20, 77). They all have Ser^G:6^ instead of the more common His^G:6^ and Ala^1^. Class 2 inteins bypass the first step of splicing with Cys^+1^ directly attacking the N-terminal splice site amide bond, resulting in the same block G branched intermediate as in class 1 inteins. Thereafter, they follow the standard splicing pathway (76, 77). How class 2 inteins activate the N-terminal splice site for direct attack by Cys^+1^ and why class 1 inteins cannot (13–15, 65) remains to be fully determined. A possible explanation comes from the NMR structure of the Mja KlbA intein where a slight widening of its active site as compared with class 1 inteins allows the Cys^+1^ nucleophile to approach the N-terminal splice site without formation of a linear (thio)ester intermediate (76). The same three residues (Thr^B:7^, His^B:10^, Asp^F:4^) that activate class 1 N-terminal splice sites are also required in class 2 inteins (76, 77). Mutation of His^B:10^ and Asp^F:4^ block splicing and drastically reduce both N-terminal and C-terminal cleavage (76, 77). Thr^B:7^ and His^B:10^ are positioned near the backbone nitrogen of Ala^1^, and modeling of an active conformation showed Asp^F:4^ hydrogen bonding to the Cys^+1^ thiol to possibly activate it by deprotonation (76).

Class 3 inteins have a remarkable mechanism that includes two branched intermediates (Fig. 4) (10, 82, 83). Cys is conserved at position F:4 in all class 3 inteins. It directly attacks the N-terminal splice site amide bond, resulting in the N-extein linked by a thioester to Cys^F:4^, yielding a block F branched intermediate. Next, the N-extein is transferred to the side chain of Cys^+1^ to form a standard block G branched intermediate. Tori *et al.* (10) hypothesized that the position of Cys^F:4^ in the intein active site allows it to substitute for the loss of the intein N-terminal nucleophile, in conjunction with two other positions that are conserved in all class 3 inteins. Monophyletic class 3 inteins appear to have arisen in a phage gene and spread to helicase genes in numerous organisms (52, 82). Thus the evidence suggests that both class 2 and class 3 inteins arose from single events.

There are at least two classes of BILs (38). Type A BILs have C-terminal His-Asn residues like inteins and can splice, although cleavage products dominate; type B BILs lack similarity to intein block G and catalyze splice site cleavage reactions uncoupled to splicing (38). The proposed mechanism for type A BIL splicing involves formation of a thioester bond at the BIL N terminus (intein step 1) and cleavage at the BIL C terminus by Asn cyclization (intein step 3). The free amino group on the C-terminal fragment attacks the N-terminal thioester bond to ligate the fragments flanking the BIL (36–38). The *Magnetospirillum magnetotacticum* BIL did not splice until Tyr^+1^ was mutated to Cys (39), suggesting that it is still tuned to act like an intein. It is likely that BILs arose in the distant past from mutated inteins or from a common ancestor of inteins.

## Regulation of Splicing by Mechanism-linked Conformational Changes and Kinetic Rates

Although the basic steps in protein splicing were elucidated in the 1990s, we still lack a consensus for how they are coordinated. Two basic processes are invoked: 1) conformational changes triggered by a preceding step result in formation of a robust active site for the next step and 2) differences in kinetic rates for each step ensure correct reaction order. Conformational changes may be as simple as fixing different rotamer positions, or they may involve larger movements. Evidence for conformational control can be inferred from the absolute coupling of N-terminal and C-terminal reactions observed in some inteins where C-terminal cleavage only occurs if preceding steps have been completed (14, 59, 69, 84, 85).

The most common argument for larger scale movement in intein active sites comes from intein structures. Only the Sce VMA intein (86) and the Pho RadA intein (46) structures have distances between the C-extein nucleophile and N-terminal scissile bond that are directly compatible with catalysis (3.8 Å). This distance is much larger (∼8 Å) in all other intein structures to date and requires a conformational change for catalysis (41). A conformational shift was also proposed in the class 2 Mja KlbA intein where a rearrangement of Ser^G:6^, Asn^G:7^, and Cys^+1^ (G:8) backbone torsional angles could enable a close approach of the Cys^+1^ nucleophile to the N-terminal scissile bond (76). It remains to be determined why inteins display such an open active site and whether it represents a true conformation or an artifact of experimental conditions that prevent splicing, including mutations to active site residues and differences in extein sequence or length.

Movement of side chains during splicing can coordinate the reaction by the gain or loss of hydrogen bonds and changes in van der Waals packing interactions to align catalytic residues. For example, structures of the Pho RadA intein suggest that Asp^F:4^ hydrogen-bonds to Asn^G:7^, preventing Asn cyclization until branched intermediate formation causes reorientation of the Asp^F:4^ side chain (46). Another example involves coupling of N- and C-terminal cleavage in the Ssp DnaE intein, which is proposed to be due to Tyr^−1^ preventing proper orientation of Arg^B:11^ until formation of the linear and/or branched thioester intermediate results in movement of the Tyr^−1^ side chain, allowing the Arg^B:11^ side chain to reorient and assist Asn cyclization (87). In the Mxe GyrA intein, NMR data show that chemical or conformational changes in the branched intermediate stimulates Asn cyclization (69).

Kinetic data can provide further insight into how inteins control the steps of splicing. Asn cyclization is the slowest step for most inteins studied to date, including the Pab Pol II intein (71), the split Ssp DnaE intein (32, 49, 88), and the Mxe GyrA intein (69). In the Pab Pol II intein, substitution of Gln^G:7^ with the more common Asn^G:7^ accelerated C-terminal cleavage by 20-fold and the overall splicing reaction by 3-fold. The naturally split Npu and Ssp DnaE inteins have been extensively investigated as model systems for intein kinetics because it is easy to initiate reactions by mixing fragments (29, 32, 48, 49, 87–90). Ssp DnaE intein studies demonstrate that association between the fragments is not rate determining (32). Whereas Asn cyclization is the slow step for the Ssp DnaE intein, all steps occur with similar rates in the Npu DnaE intein (32, 49). Although the Ssp DnaE intein splices with overall rates similar to standard inteins, the Npu DnaE intein splices very rapidly, with a half-life of 1 min or less (30, 89). Recently discovered split inteins from metagenomic samples can splice even more rapidly (91).

The class 2 Mja KlbA intein was studied using a semisynthetic intein precursor that could be induced to splice with a redox switch (92). Branched intermediate formation was comparable with the rate of Asn cyclization, and a clear rate-limiting step was not identified.

In all of these inteins, the rates of Asn cyclization are comparable or slower than preceding steps, ensuring that Asn cyclization will not precede extein ligation, which occurs during branched intermediate formation. Kinetic control complements coordination strategies involving conformational changes. This was shown experimentally in the Mxe GyrA intein, where the rate of C-terminal cleavage increased 10-fold when a branched intermediate was present (69).

## Conditional Protein Splicing

Inteins have evolved to tightly regulate the steps of splicing. This is essential as inteins interrupt highly conserved domains of proteins important to their host organisms, including DNA polymerases and helicases (93). However, no evidence has been discovered for a physiologically relevant role for conditional protein splicing. This suggests that modern inteins are likely molecular parasites and that efficient, traceless splicing is essential for their maintenance in the host genome. However, inteins can be engineered to be sensitive to changes in light, pH, temperature, or redox state and to be responsive to the addition of small molecules (33, 94). Even unmodified inteins can be controlled under specific conditions. For example, inteins from thermophilic organisms display temperature-dependent splicing in heterologous precursors (7, 78, 95), both *cis*-splicing and *trans*-splicing inteins are sensitive to inhibition by divalent cations (87, 88, 96–98), and disulfide bonds involving active site Cys residues sensitize splicing to cellular oxidation state (79, 99, 100).

## Conclusions

Remaining mechanistic challenges include deciphering how reactions are coordinated and illuminating the diverse ways that inteins promote catalysis. Going forward, detailed studies of catalytic mechanisms, intein kinetics, and structures must occur in the context of native host exteins, which will distinguish between physiologically significant observations and those that may be artifacts of heterologous model systems. Furthermore, detailed studies of multiple inteins will determine whether catalytic strategies are universal or specific to a subset of inteins.

The plethora of reactions performed by HINT domain proteins highlights the robust and flexible nature of catalysis when rapid turnover and substrate binding are not required. This allows for survival of mutated inteins as long as compensatory residues are present to permit a low level of splicing and provides time for the intein to evolve into a more efficient enzyme by testing new catalytic strategies. Thus the flexibility of inteins, BILs, and Hedgehog proteins provides a blueprint for modifying enzyme activity by varying nucleophiles and strategies to activate these nucleophiles.

## Footnotes

2 The abbreviations used are:

BIL: bacterial intein-like
Pol: polymerase.
